# Global burden of pelvic inflammatory disease and ectopic pregnancy from 1990 to 2019

**DOI:** 10.1186/s12889-023-16663-y

**Published:** 2023-10-02

**Authors:** Deng He, Tian Wang, Wu Ren

**Affiliations:** Department of Obstetrics and Gynecology, Tongji Hospital, Tongji Medical College, Huazhong University of Science and Technology, Wuhan, Hubei People’s Republic of China

**Keywords:** Pelvic inflammatory disease, Ectopic pregnancy, Global burden of disease, Prevalence, Incidence, Correlation

## Abstract

**Background:**

Pelvic inflammatory disease (PID) is a widespread female public problem worldwide. And it could lead to infertility, preterm labor, chronic pelvic pain, and ectopic pregnancy (EP) among reproductive-aged women. This study aimed to assess the global burden and trends as well as the chaning correlation between PID and EP in reproductive-aged women from 1990 to 2019.

**Methods:**

The data of PID and EP among reproductive-aged women (15 to 49 years old) were extracted from the Global Burden of Disease study 2019. The disease burden was assessed by calculating the case numbers and age-standardized rates (ASR). The changing trends and correlation were evaluated by calculating the estimated annual percentage changes (EAPC) and Pearson’s correlation coefficient.

**Results:**

In 2019, the ASR of PID prevalence was 53.19 per 100,000 population with a decreasing trend from 1990 (EAPC: − 0.50), while the ASR of EP incidence was 342.44 per 100,000 population with a decreasing trend from 1990 (EAPC: − 1.15). Globally, PID and EP burdens changed with a strong positive correlation (Cor = 0.89) globally from 1990 to 2019. In 2019, Western Sub-Saharan Africa, Australasia, and Central Sub-Saharan Africa had the highest ASR of PID prevalence, and Oceania, Eastern Europe, and Southern Latin America had the highest ASR of EP incidence. Only Western Europe saw significant increasing PID trends, while Eastern Europe and Western Europe saw increasing EP trends. The highest correlations between PID and EP burden were observed in Burkina Faso, Laos, and Bhutan. General negative correlations between the socio-demographic index and the ASR of PID prevalence and the ASR of EP incidence were observed at the national levels.

**Conclusion:**

PID and EP continue to be public health burdens with a strong correlation despite slightly decreasing trends detected in ASRs globally. Effective interventions and strategies should be established according to the local situation by policymakers.

**Supplementary Information:**

The online version contains supplementary material available at 10.1186/s12889-023-16663-y.

## Introduction

Pelvic inflammatory disease (PID) is an inflammatory process of the upper genital tract, including the uterus, fallopian tubes, and related pelvic organs, which is mainly caused by sexually transmitted infections (STIs) [1, 2]. PID could lead to salpingitis and tubal adhesion which increase the possibility of ectopic pregnancy (EP) among reproductive-aged women. During the 1960s to 1970s, due to gonorrhea infection, increasing PID incidence was observed in many developed countries [3]. However, with the implementation of measures to control sexually transmitted infections (STIs) and changes in sexual behavior prompted by the threat of AIDS, many countries worldwide have reported declining rates of PID [4–12]. And many high-income countries sustained to launch programs to screen and treat women for asymptomatic STIs to prevent PID [13–16].

Limited current data exists on the epidemiology of PID across the globe. The National Health and Nutrition Examination Survey 2013–2016 and National Survey of Family Growth 2015–2017 reported the self-reported history of PID in women aged 18–44 years was 4.1% (95% CI, 3.2%–5.1%) and 3.6% (95% CI, 2.9%–4.5%) in America, respectively [17, 18]. These studies have also shown an overall decline in self-reported PID history, except for small increases in 2015 [18]. Similarly, a national data set from England spanning from 2009 to 2019 has observed a decline in the diagnosis of PID over the decade. Specifically, chlamydial PID has declined by 58%, gonococcal PID by 34%, and "nonspecific" PID by 37% [19]. These studies are all from high-income populations; for most populations in low- and middle-income countries, epidemiological research on PID and its sequelae is lacking.

Women with PID are far more likely to have infertility, chronic pelvic pain, and EP than women without PID [20]. A follow-up study conducted on Swedish women who received laparoscopy and treatment for clinically suspected PID revealed that the salpingitis rate was 74%, with 16% of women with confirmed salpingitis experiencing infertility and 9% experiencing a subsequent EP [20]. The PID Evaluation and Clinical Health (PEACH) trial found that after a 3-year follow-up, approximately 29% of participants experienced chronic pelvic pain, 18% experienced infertility, and 0.6% experienced EP [21]. It is important to note that delayed treatment for PID has been strongly associated with worse long-term reproductive outcomes. PID has been linked to spontaneous abortion, chorioamnionitis, pre-term premature rupture of membranes, preterm labor, and stillbirth [22, 23]. As a better reproductive outcome is an important issue for women, families and society, the need for preventing PID and its sequalae impact is urgent.

In addition to sexually transmitted organisms, there are two other general groups of pathogens that have been associated with PID: bacterial vaginosis-associated bacteria and gastrointestinal or respiratory bacteria [24]. In studies from high-income population, bacterial vaginosis–associated microbes have been implicated as potential causes [25]. And a clinical trial on the addition of metronidazole to ceftriaxone and doxycycline was proved to be effective on acute PID [26]. The National Institutes of Health has convened a workshop to identify research needs for the improvement of the diagnosis, treatment, and prevention of pelvic inflammatory disease, which emphasized screening test and biomarker development [27]. But for financial and logistic reasons, PID prevention programs that are based on screening test are not feasible in most low-income and middle-income countries, where the clinicians rely on syndromic management [2].

The global burden of PID is probably substantial, and the prevention is of urgent need. But the epidemiological situations may have changed with slight changes in pathogens and differ according to socio-economic development in some countries. To accurately assess the impact of public health initiatives such as STI screening and treatment on the prevalence of PID, it is necessary to conduct high-quality and current studies. Additionally, it is crucial to gather data that can pinpoint the sources of regional and national disparities in the prevalence of PID and its consequential effects, in order to help policymakers and clinicians to develop interventions. The Global Burden of Disease (GBD) study has yearly estimated a range of disease metrics since 1990, which offered a fantastic chance for comparative evaluation of disease burden and trends in the 204 global countries and territories [28, 29]. Therefore, using the data from the GBD 2019, we aimed to estimate the burden, changing trends, and correlations between PID and EP for reproductive-aged women in 204 countries and territories during the period of 1990 to 2019, which would provide meaningful information about secular trends and gaps from comparisons of PID and EP burden and changing patterns across various nations and areas.

## Methods

### Study data source

The GBD Study conducted by the Institute for Health Metrics and Evaluation is a resource that measures epidemiological levels and trends comprehensively and comparably across the world. The GBD 2019 performed a comparative assessment of health losses and associated risk factors for 369 diseases and injuries in 204 countries and territories from 1990 to 2019 using all of the most recent and available sources of epidemiological survey data and optimized, standardized methods. The Global Health Data Exchange (GHDx) query tool (http://ghdx.healthdata.org/gbd-results-tool) was used to extract all used data from GBD 2019. This study assessed the PID and EP burden of women within reproductive age (15 to 49 years old) using measures of prevalence and incidence in 204 countries and territories within 21 geographic regions between 1990 and 2019. The estimates and their 95% uncertainty intervals (UIs) were extracted for the prevalence of PID and the incidence of EP. Additionally, data on socio-demographic index (SDI), an assessment of the development of each geographic entity, was gathered for different nations and areas. The SDI ranges from 0 to 1.0 and divides countries and territories into five quintiles: low (< 0.455), low-middle (0.455—0.608), middle (0.608- 0.690), high-middle (0.690–0.805), and high (0.805–1.0) value regions. The cut-off values used to determine quintiles for analysis were computed using country-level estimates of the SDI for the year 2019, excluding countries with populations of less than 1 million.

### Statistical analysis

To compare populations with different age structures or the same population with changing age structures in different time periods, data were standardized by applying age-standardized rate (ASR). The ASR did not reflect the actual case number, but was only used to compare the burden in different countries, different regions, or different historical periods in the same region, so as to facilitate data comparisons. In this study, ASR and case numbers were calculated to reflect the disease burden, while the estimated annual percentage changes (EAPC) were calculated to describe the changing trends. We used the GBD world population age standard to calculate the age-standardized rate in reproductive-aged women (15 to 49 years old) [30]. The ASRs, including age-standardized prevalence rates (ASPRs) and age-standardized incidence rates (ASIRs) were estimated using the following formula:

$$\mathrm{ASR}=\frac{{\sum }_{i=1}^{A}{a}_{i}{w}_{i}}{{\sum }_{i=1}^{A}{w}_{i}}\times \mathrm{100,000}$$

where, in the i^th^ age group, a_i_ denotes the age-specific rate, w denotes the number of individuals (or weight) in that age group from the chosen standard population, and A denotes the total number of age groups.

The EAPC is a frequently used statistic to describe the changing trend of ASRs. A regression line was fitted to the natural logarithm of the rates (ASR). EAPC and its 95% confidence interval (CI) were estimated using this linear model. The formulae were as follows:

$$\begin{array}{c}\mathrm{y}=\mathrm{\alpha }+\mathrm{\beta w}+\upvarepsilon \\ \mathrm{EAPC}=100\times (\mathrm{exp}\left(\upbeta \right)-1)\end{array}$$

where y = ln (ASR) and x = calendar year. Trends were assessed as follows: 1. ASR was considered to hold an increasing trend with EAPC and its 95% CI > 0; 2. ASR was considered to hold a decreasing trend with EAPC and its 95% CI < 0.

Changing correlation between ASPR of PID and ASIR of EP were evaluated using the Pearson’s correlation test model. In this study, data analysis was performed on R program (Version 3.6.2), and a *p*-value of < 0.05 was considered to be statistically significant.

## Result

### Global burden and trends of PID and EP

**Fig. 1:**
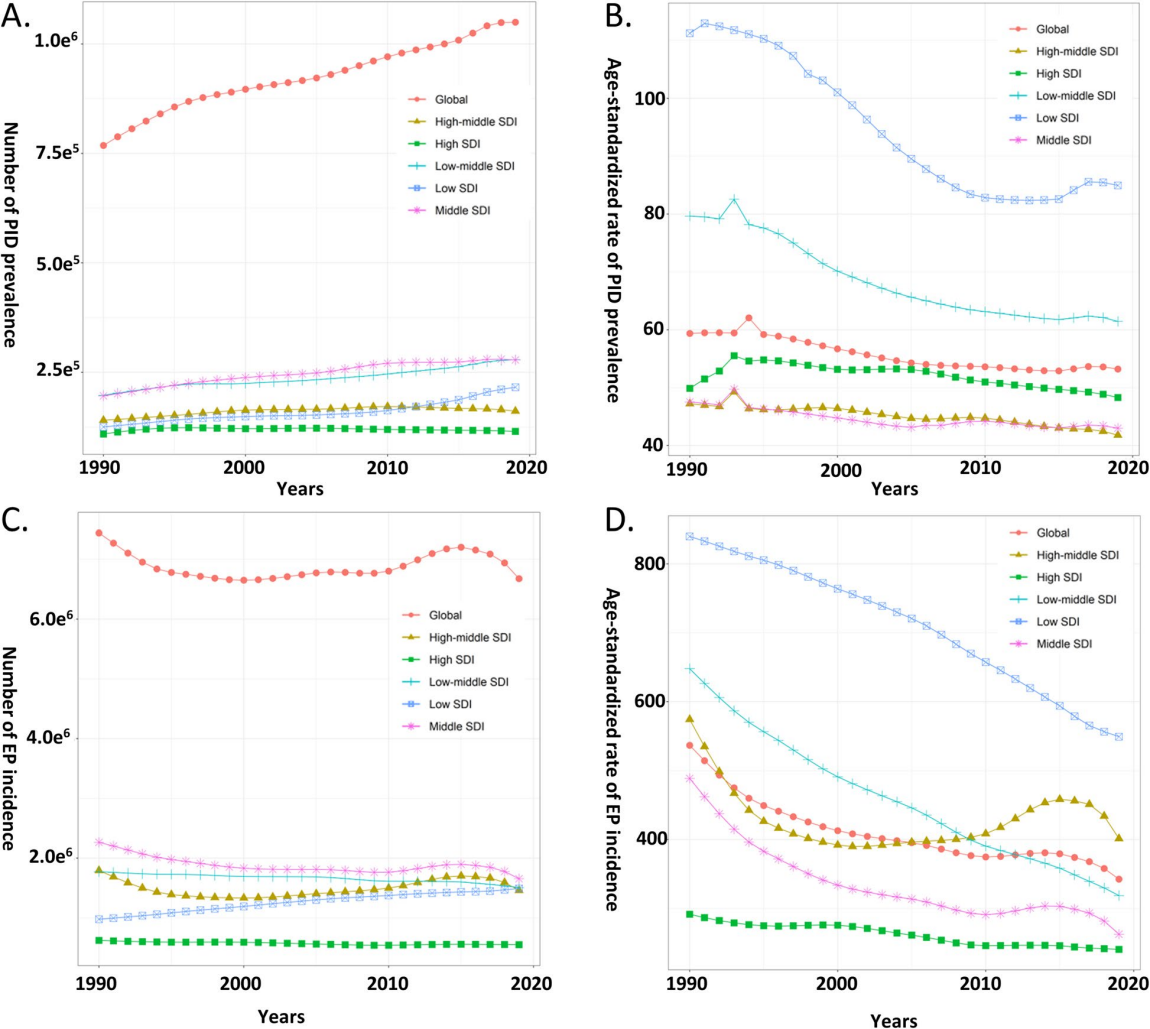
The Burden of Global Pelvic Inflammatory Disease (PID) and Ectopic Pregnancy (EP) by Different SDI Regions from 1990 to 2019; **A**) The PID prevalence number, **B**) The age-standardized prevalence rates of PID, **C**) The EP incidence number, **D**) The age-standardized incidence rates of EP

In 2019, there were 1.05 million reproductive-aged women holding active PID globally (95% UI: 0.77 to 1.43 million), with an ASPR of 53.19 per 100,000 people (95% UI: 33.02 to 88.61) (Fig. 1A and B). The prevalence number of PID increased by 36.66% (95% UI: 31.76–41.16%) and the ASPR slightly decreased (EAPC: − 0.50, 95% CI − 0.42 to -0.58) from 1990 to 2019 (Fig. 1A and Table 1). Concurrently, the number of incidence cases of EP for reproductive women was 6.68 million (95% UI: 5.21 to 8.58 million) in 2019, and the ASIR was 342.44 per 100,000 population (95% UI: 223.42 to 495.14) (Fig. 1C and D). The incidence cases decreased by 10.26% (95% UI: -16.00 – -4.07%) and the ASIR decreased (EAPC: − 1.15, 95% CI: − 1.32 to -0.98) from 1990 to 2019 (Fig. 1C and Table 1). Almost all SDI areas held decreasing trends in ASR of PID and EP, and the low SDI area had the highest ASPR of PID and ASIR of EP during the study period (Fig. 1B and D).

**Table 1:** The Burden and Trends of Pelvic Inflammatory Disease and Ectopic Pregnancy in Reproductive-aged Women from 1990 to 2019

Characteristics	ASPR of PID in 2019 (95% UI)	EAPC of ASPR from 1990 to 2019 (95% CI)	ASIR of EP in 2019 (95% UI)	EAPC of ASIR from 1990 to 2019 (95% CI)
**Global**	53.19(33.02–80.61)	-0.50(-0.58–0.42)	342.44(223.42–495.14)	-1.15(-1.32–0.98)
**SDI**				
High SDI	48.28(31.11–71.07)	-0.33(-0.44–0.22)	240.48(167.72–333.04)	-0.66(-0.72–0.6)
High-middle SDI	41.77(25.69–63.5)	-0.40(-0.45–0.34)	401.33(265.3–571.81)	-0.31(-0.71–0.1)
Middle SDI	42.98(26.55–65.62)	-0.35(-0.44–0.27)	262.58(171.6–379.14)	-1.59(-1.86–1.32)
Low-middle SDI	61.44(37.56–93.84)	-1.05(-1.17–0.92)	318.56(202.39–463.06)	-2.29(-2.34–2.24)
Low SDI	84.95(53.21–128.74)	-1.29(-1.47–1.11)	549.25(342.12–816.9)	-1.48(-1.57–1.38)
**Region**				
Andean Latin America	67.19(43.79–99.43)	-0.47(-0.51–0.43)	534.37(345.33–785.65)	-1.26(-1.41–1.12)
Australasia	93.67(57.01–146.17)	-0.29(-0.31–0.26)	132.36(91.72–184.29)	-0.60(-0.97–0.24)
Caribbean	53.35(32.33–82.19)	-0.39(-0.56–0.22)	282.(173.13–423.83)	-0.78(-0.87–0.69)
Central Asia	56.83(34.21–88.41)	-0.37(-0.44–0.3)	463.66(292.51–691.25)	-0.65(-1.01–0.29)
Central Europe	43.77(27.68–65.25)	-0.38(-0.46–0.29)	236.41(156.8–335.69)	-0.35(-0.72–0.03)
Central Latin America	37.82(23.16–57.59)	-0.51(-0.61–0.41)	306.16(199.48–449.4)	-1.88(-2.06–1.7)
Central Sub-Saharan Africa	83.49(51.49–126.85)	-0.67(-0.77–0.57)	566.91(343.79–859.66)	-1.30(-1.5–1.1)
East Asia	34.2(21.22–51.45)	-0.47(-0.69–0.25)	386.56(253.85–553.05)	-0.87(-1.38–0.37)
Eastern Europe	48.27(28.8–75.54)	-0.64(-0.85–0.43)	705.23(449.15–1049.92)	1.44(0.82–2.07)
Eastern Sub-Saharan Africa	74.29(45.69–113.95)	-1.28(-1.5–1.06)	542.41(332.54–810.94)	-1.57(-1.66–1.48)
High-income Asia Pacific	80.56(49.45–122.26)	0.05(-0.02–0.12)	124.29(85.23–174.74)	-0.71(-0.91–0.52)
High-income North America	50.85(35.15–70.39)	-0.76(-1.–0.52)	160.51(121.44–210.6)	-2.36(-2.8–1.92)
North Africa and Middle East	59.50(35.56–93.35)	-0.38(-0.46–0.3)	271.59(172.32–401.36)	-2.00(-2.14–1.87)
Oceania	78.55(51.4–115.03)	-1.17(-1.4–0.94)	759.01(467.13–1134.26)	-0.46(-0.47–0.44)
South Asia	63.02(38.4–96.39)	-1.01(-1.13–0.89)	274.66(171.99–403.43)	-2.68(-2.72–2.63)
Southeast Asia	17.77(10.92–27.19)	-0.61(-0.7–0.53)	211.00(135.46–310.47)	-1.44(-1.49–1.38)
Southern Latin America	65.50(39.01–103.29)	-0.58(-0.64–0.53)	676.93(422.55–1014.75)	-0.83(-0.93–0.73)
Southern Sub-Saharan Africa	74.50(45.44–115.24)	-1.56(-2.05–1.06)	163.54(102.87–240.56)	-1.49(-1.59–1.38)
Tropical Latin America	51.44(32.52–76.68)	-1.39(-1.71–1.06)	171.45(111.15–251.34)	-0.96(-1.08–0.85)
Western Europe	27.45(16.85–41.95)	0.69(0.35–1.04)	365.00(241.82–521.34)	0.68(0.58–0.79)
Western Sub-Saharan Africa	116.02(71.98–175.84)	-1.95(-2.23–1.67)	599.39(368.76–897.25)	-1.21(-1.31–1.11)

^*^ASPR Age-standardized prevalence rate, *PID* Pelvic inflammatory disease, *ASIR* Age-standardized incidence rate, *EP* Ectopic pregnancy, *SDI* Socio-demographic index, *UI* Uncertain interval, *CI* Confidential interval

### Pelvic inflammatory disease burden at the regional and national level

In 2019, the highest ASPR of PID per 100,000 population for reproductive-aged women were observed in Western Sub-Saharan Africa (116.02 (95% UI: 71.98 to 175.84)), Australasia (93.67 (95% UI: 57.01 to 146.17)), and Central Sub-Saharan Africa (83.49 (95% UI: 51.49 to 126.85)), while the lowest rates were observed in Southeast Asia (17.77 (95% UI: 10.92 to 27.19)), Western Europe (27.45 (95% UI: 16.85 to 41.95) and East Asia (34.20 (95% UI: 21.22 to 51.45)) (Table 1). Statistically significant increases for ASPR from 1990 to 2019 was only observed in Western Europe (EAPC: 0.69 (95% CI: 0.35 to 1.04)), and most regions held decreasing changes, especially in Western Sub-Saharan Africa (EAPC: -1.95 (95% CI: -2.23 to -1.95)), Southern Sub-Saharan Africa (EAPC: -1.56 (95% CI: -2.05 to -1.06)), and Tropical Latin America (EAPC: -1.39 (95% CI: -1.71 to -1.06)) (Table 1).

**Fig. 2:**
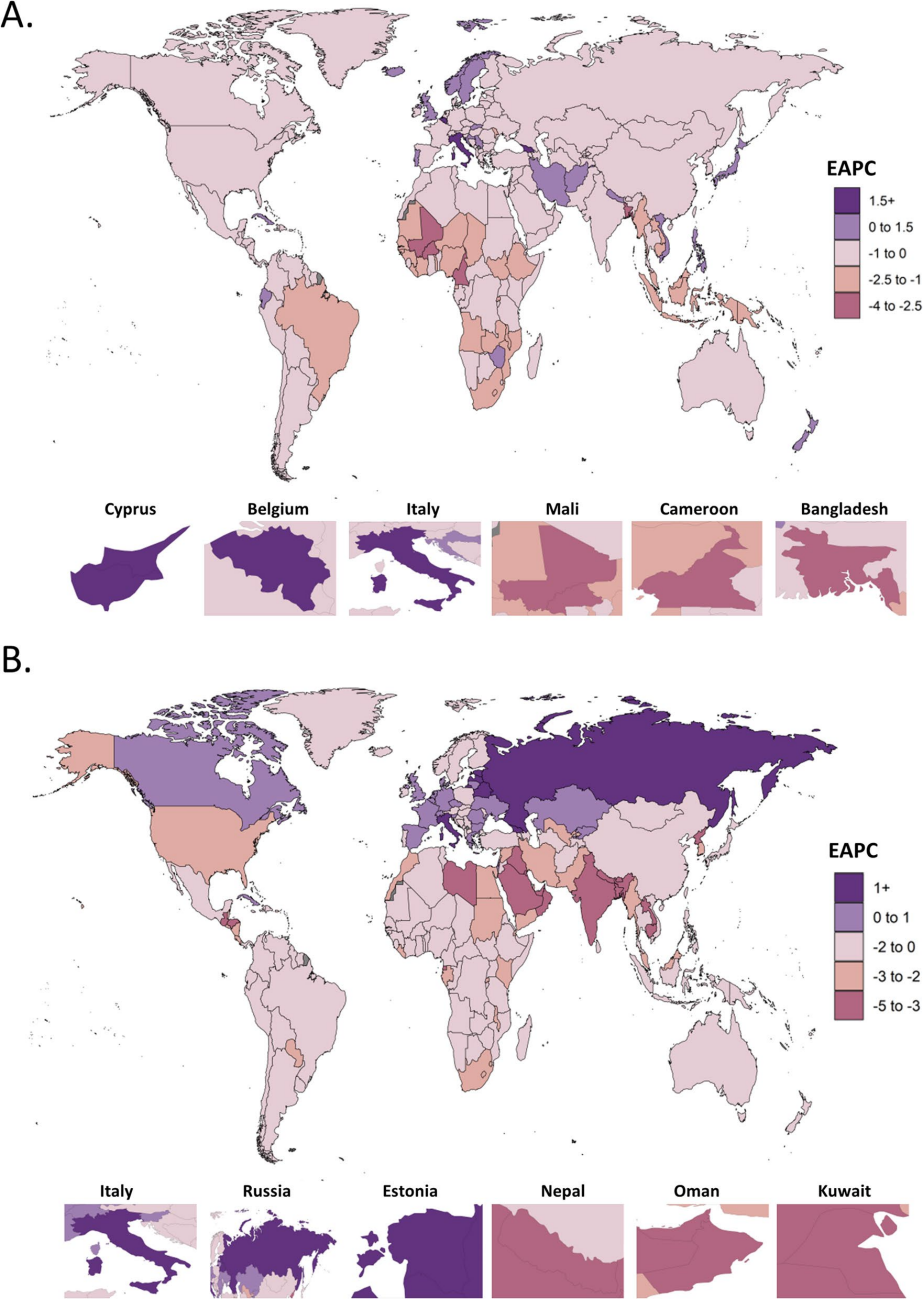
The Estimated Annual Percentage Changes (EAPCs) at the National Level, 1990–2019. **A**) EAPC of age-standardized prevalence rates for pelvic inflammatory disease from 1990 to 2019, **B**) EAPC of age-standardized incidence rates for ectopic pregnancy from 1990 to 2019

At the national level, the highest ASPRs in 2019 were observed in the Low SDI countries of Niger (133.56 (95% UI: 82.82 to 204.13)), Burkina Faso (133.20 (95% UI: 83.07 to 199.69)), and Gambia (133.06 (95% UI: 81.56 to 204.25)), with the lowest rates occurred in the High and High-middle SDI countries of Iceland (5.17 (95% UI: 2.63 to 9.07)), Cyprus (6.85 (95% UI: 3.38 to 12.08)), and Portugal (6.94 (95% UI: 4.12 to 10.83)) (Table S1). From 1990 to 2019, the changing trends of ASPR varied differently according to countries, with the pronounced increasing change observed in the High and High-middle SDI countries of Cyprus (EAPC: 2.04(95% CI: 0.94 to 3.15)), Belgium (EAPC: 1.88 (95% CI: 0.35 to 3.43)), and Italy (EAPC: 1.85 (95% CI: 1.22 to 2.48)), while the deepest decreasing changes were recorded in the Low and Low-middle SDI countries of Mali (EAPC: -3.51 (95% CI: -3.93 to -3.09)), Cameroon (EAPC: -2.63 (95% CI: -2.95 to 2.30), and Bangladesh (EAPC: -2.55 (95% CI: -3.04 to -2.05)) (Fig. 2A and Table S1).

### Ectopic pregnancy burden at the regional and national level

The highest ASIR of EP for reproductive-aged women in 2019 were observed in Oceania (759.01 (95% UI: 467.13–1134.26)), Eastern Europe (705.23 (95% UI: 449.15–1049.92)), and Southern Latin America (676.93 (95% UI: 422.55–1014.75)), while High-income Asia Pacific (124.29 (95% UI: 85.23–174.74)), the Australasia (132.36 (95% UI: 91.72–184.29)) had the lowest rates, and High-income North America (160.51 (95% UI: 121.44–210.6)) (Table 1). Statistically significant increasing trends for ASIR from 1990 to 2019 were only observed in Eastern Europe (EAPC: 1.44 (95% CI: 0.82 to 2.07)) and Western Europe (EAPC: 0.68 (95% CI: 0.58 to 0.79)), and most regions held decreased changes, especially in South Asia (EAPC: -2.68 (95% CI: -2.72 to -2.64)), High-income North America (EAPC: -2.36 (95% CI: -2.80 to -1.92)), and North Africa and Middle East (EAPC: -2.00 (95% CI: -2.14 to -1.87)) (Table 1).

At the national level, the highest ASIRs in 2019 were observed in the Low SDI countries of Niger (871.33 (95% UI: 521.99–1352.27)) and Somalia (835.61 (95% UI: 504.06–1262.06)), and the High SDI country of Austria (835.8 (95% UI: 539.64–1236.73)), while the lowest rates occurred in the High SDI countries of Australia (60.65 (95% UI: 42.26–84.01)) and South Korea (87.95 (95% UI: 71.66–107.69)), and the Middle SDI country of South Africa (75.01 (95% UI: 46.84–111.16)) (Table S1). From 1990 to 2019, the changing trends of ASIR also varied differently according to countries. The High and High-middle SDI countries of Italy (EAPC: 1.67 (95% CI: 1.29 to 2.06)), Russia (EAPC: 1.65 (95% CI: 0.96 to 2.33)), and Estonia (EAPC: 1.30 (95% CI: 0.82 to 1.78)) held the most pronounced increasing trends, while the deepest decreasing trends were reported in the Low SDI country Nepal (EAPC: -4.35 (95% CI: -4.64 to -4.06)), the High-middle SDI country Oman (EAPC: -4.30 (95% CI: -4.55 to -4.06)), and the High SDI country Kuwait (EAPC: -3.89 (95% CI: -4.75 to -3.03)) (Fig. 2B and Table S1).

### Association between prevalence of PID and incidence of EP

**Fig. 3:**
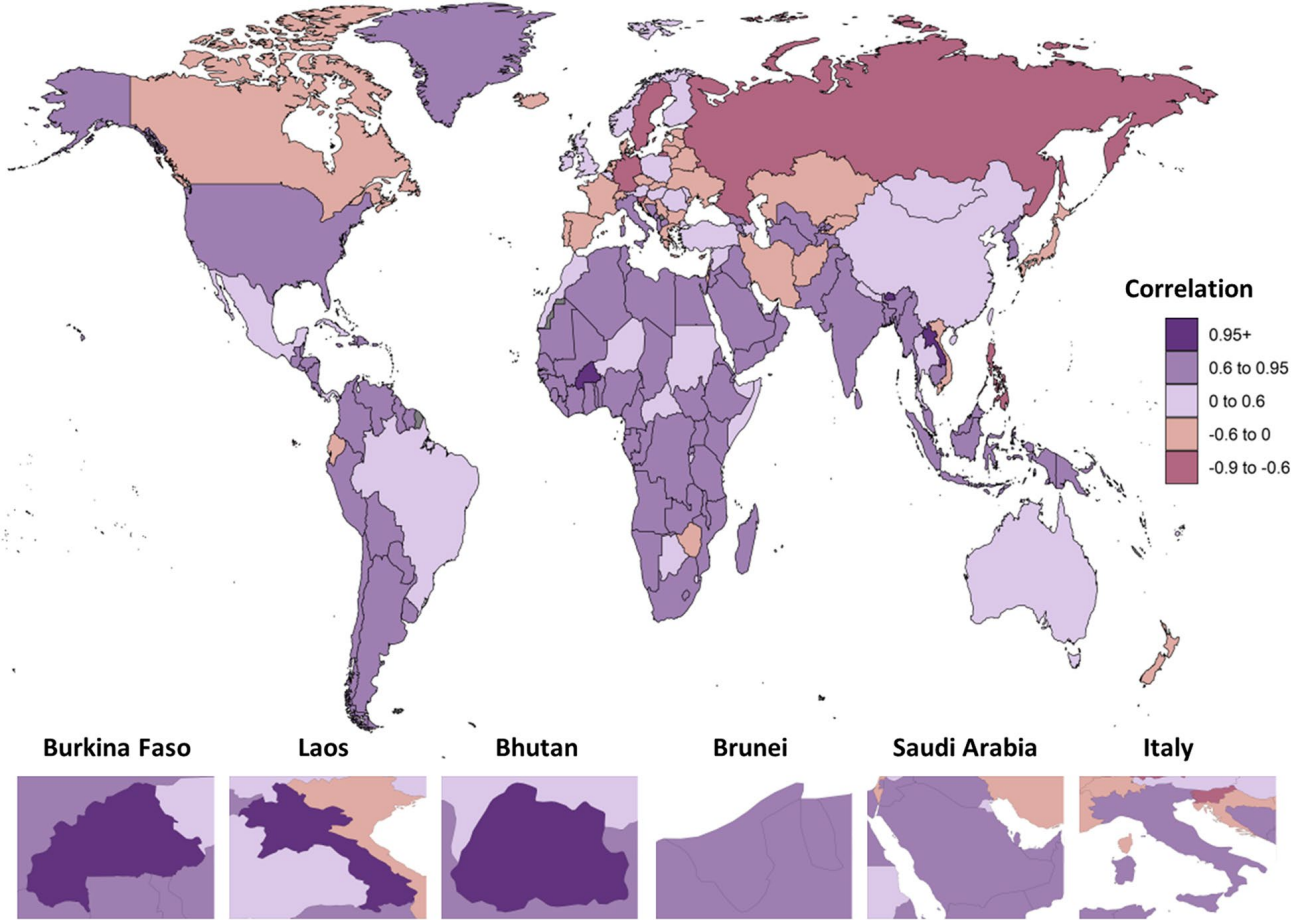
Correlation Coefficient between Age-standardized Prevalence Rates for Pelvic Inflammatory Disease and Age-standardized Incidence Rates for Ectopic Pregnancy at the national level from 1990 to 2019

The majority of countries (158 out of 204, or 77.45%) have exhibited a decreasing trend in both the ASPR of PID and the ASIR of EP since 1990. However, a handful of countries, namely Italy, Belgium, Georgia, Cuba, and the UK, have displayed an increasing trend in both ASPR of PID and ASIR of EP during the same period (Table S1). At the global level, a positive correlation between ASPR of PID and ASIR of EP has been observed from 1990 to 2019 (Cor = 0.89, *p*-value < 2.2e-16) (Fig. S1A). Furthermore, a correlation coefficient has been calculated at the national level, and 37 countries showed a correlation value higher than the global level. These countries include two from the High SDI quintile, three from the High-middle SDI quintile, six from the Middle SDI quintile, 16 from the Low-middle SDI quintile, and 10 from the Low SDI quintile. Notably, Burkina Faso, a Low SDI country, has exhibited the highest coefficient value (Cor = 0.96, *p*-value < 2.2e-16), followed by Laos (Cor = 0.95, *p*-value < 2.2e-16) and Bhutan (Cor = 0.95, *p*-value < 2.2e-16), both Low-middle SDI countries (Fig. S1B-D). Additionally, several High SDI countries, including Brunei and Saudi Arabia, as well as High-middle SDI countries such as Oman, Dominica, and Italy, have also shown high coefficient values exceeding 0.89 (Fig. 3, Fig. S1E-I, and Table S1).

### Association between burdens and SDI index

**Fig. 4:**
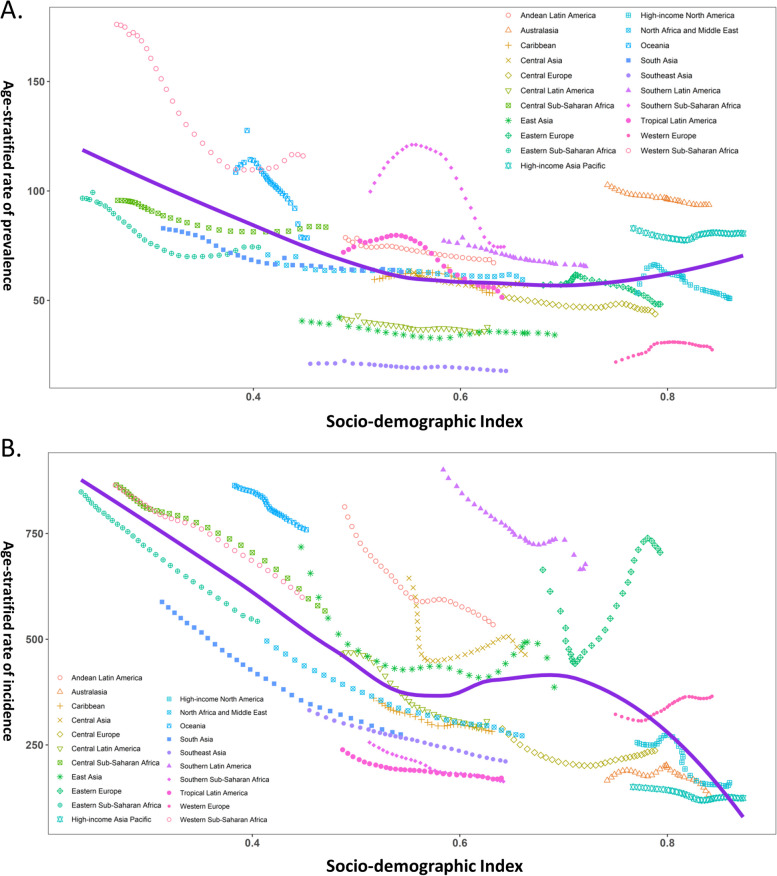
The Distribution of Age-standardized Rate (ASR) of Pelvic Inflammatory Disease Prevalence and Ectopic Pregnancy Incidence in 21 Regions from 1990 to 2019 according to Socio-demographic Index (SDI). **A**) ASR of pelvic inflammatory disease prevalence in 21 regions by SDI, **B**) ASR of ectopic pregnancy incidence in 21 regions by SDI

In analyzing the relationship between the ASPR of PID and SDI across various geographic regions, a trend line was fitted whose bottom was between SDI values of 0.6 and 0.8 before increasing with increasing SDI values, as illustrated in Fig. 4A. From 1990 to 2019, several regions including Western Sub-Saharan Africa, Oceania, Southern Sub-Saharan Africa, Andean Latin America, Southern Latin America, Australia, and High-income Asia Pacific had higher ASPRs than expected given their levels of socio-demographic development. Conversely, the burdens of Eastern Sub-Saharan Africa, East Asia, Central Latin America, Southeast Asia, Central Europe, and Western Europe were all lower than anticipated during the measurement period.

At the regional level, the ASIR of EP and the SDI index were fitted by a general decreasing trend line except for SDI values between 0.6 and 0.7, indicating that the incidence burden of EP decreased with rising SDI values (Fig. 4B). However, it is noteworthy that the ASIRs of certain regions such as Central Sub-Saharan Africa, Western Sub-Saharan Africa, Central Asia, Oceania, Andean Latin America, South Latin America, and Eastern Europe were higher than anticipated from 1990 to 2019. On the other hand, Eastern Sub-Saharan Africa, South Asia, North Africa and Middle East, Caribbean, Southeast Asia, Southern Sub-Saharan Africa, Tropical Latin America, Central Europe, and Australasia had lower than expected burdens.

**Fig. 5:**
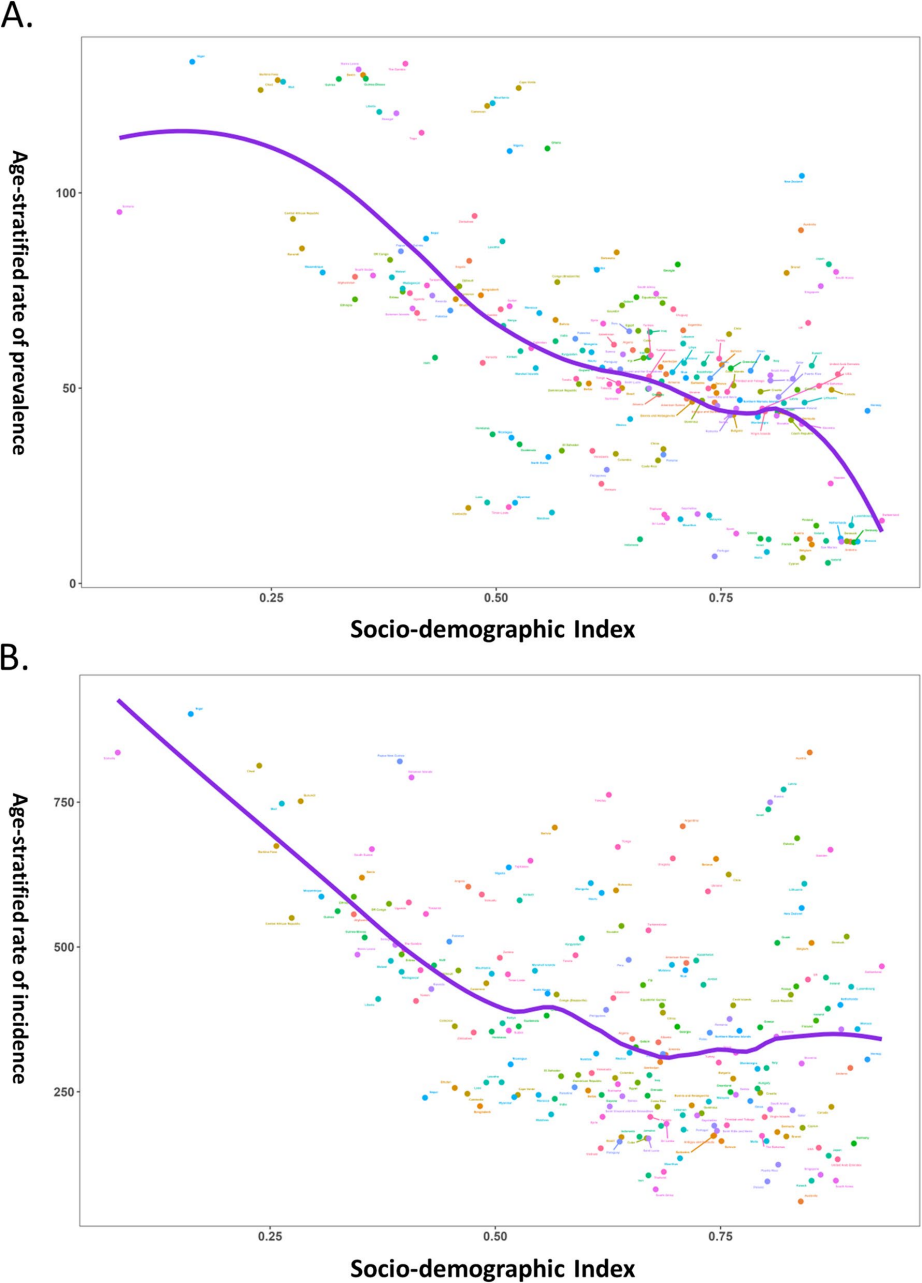
Age-standardized rates (ASR) of Pelvic Inflammatory Disease Prevalence and Ectopic Pregnancy Incidence according to Socio-demographic Index (SDI) in 204 Countries and Territories. **A**) ASR of pelvic inflammatory disease prevalence for 204 countries and territories by SDI in 2019; **B**) ASR of ectopic pregnancy incidence for 204 countries and territories by SDI in 2019

At the national level, in 2019, the ASRs of PID prevalence and EP incidence according to SDI were analyzed. The expected pattern was nonlinear for ASPR of PID and its association with SDI, but a general decreasing trend with an increasing SDI value was observed (Fig. 5A). Interestingly, countries with high SDI, such as Switzerland, Norway, and South Korea, had higher than expected PID burdens, while countries with low SDI, such as Niger, Chad, and Burkina Faso, also had higher than expected PID burdens. On the other hand, the expected pattern for ASIR of EP and its association with SDI was nonlinear in nature, with a bottom at an SDI value of approximately 0.70 before slightly increasing ASIR of EP with an increasing SDI value (Fig. 5B). High SDI countries such as Switzerland, Monaco, and Netherlands had higher than expected burdens, while low SDI countries such as Niger, Chad, and Mali also held higher than expected burdens.

## Discussion

This study estimated the substantial burden of PID and EP among reproductive-aged women worldwide over the past three decades. Globally, the prevalence cases of PID increased, while the incidence of EP for reproductive-aged women decreased from 1990 to 2019. Across the 204 countries and territories, large majority of countries displayed a downward trend in both ASPR of PID and ASIR of EP for reproductive-aged women during this period. And the ASPR of PID and ASIR of EP decreased in a positive correlation manner from 1990 to 2019, globally. Despite a decreasing trend around the world, disparities exist for regions and countries. Our study would have implications for public health officials and policymakers in different countries to implement interventions to deal with these problems. Further, by analyzing the burden and changing trends, the clinicians would be able to better understand the disease association and identify specific risk factors and pathogens.

The increased burden of active PID prevalent cases may be related to global population growth, whereas the decreased ASPR of PID reflected an improvement in medical prevention, management, and treatment. These decreases coincide with public health initiatives to control STIs, particularly *Chlamydia trachomatis* and *Neisseria gonorrhoeae* infections, whose incidence rates also exhibited declining trends from 1990 to 2019 [6, 10, 31, 32]. Furthermore, comprehensive sex education and condom promotion also contributed to the decrease in PID. According to data from the PEACH study, using condoms consistently during follow-up was linked to lower chances of PID and infertility [33]. The lowering burden of EP may coincide with the declining PID rate because the infection-induced selective loss of ciliated epithelial cells along the fallopian tube epithelium could hamper ovum transport and result in EP [2, 34]. In addition to that, condom use was also associated with a reduction in EP risk [35].

Regionally, although the highest ASPRs of PID were observed in Sub-Saharan Africa regions in 2019, which were mainly attributed to inadequate health care and sex education, these regions also had the sharpest decline since 1990, demonstrating recent advancements in PID and STI detection, diagnosis, and treatment [32, 36]. And Niger, Burkina Faso, and Gambia from these regions held the highest ASPR of PID in 2019, while Mali and Cameroon from these regions held the deepest decreasing trends of PID. In Western Europe, the low rates of PID can be attributed to the high-level medical care system. Although the rate of PID in Western Europe has slightly improved contrary to the global trend, this can be mainly attributed to Cyprus, Belgium, and Italy. According to the data, Cyprus and Belgium had much lower rates of PID in 2019 than the global level, while Italy had a higher rate than the global level. As the incidence rate of chlamydia infection in Italy women has been reported to increase significantly from 2.9 to 7.1 per 100 000 from 2005 to 2016, it has a lasting promotion effect on PID [37]. And Italy also held the highest increasing trend of EP among all the countries. Eastern Europe had the second-highest incidence rate of EP, and the greatest rising trend. This can be attributed to Estonia and Russia, where the less use of contraception methods may partly lead to an increasing number of unintended pregnancies, including EP. Reports indicate that just 52% of Russian women utilize modern contraception methods, which is lower than the estimated global average of 57%. As few as 10% of reproductive-aged women treated by gynecologists in Russia use hormonal contraception [38].

As positive correlation between PID and EP burden was found globally from 1990 to 2019, some countries stood out because of higher correlations, revealing that EP occurrence had a strong association with PID. Although there were many risk factors for EP, PID may have been one of the main causes of EP in these countries [39]. Burkina Faso from the Low SDI region, which got the second highest ASPR of PID in 2019, had the highest coefficient value. This may have a strong association with its poorer social-economic development. Italy from the High-middle SDI areas, had high coefficient values of 0.89, and this result was in consist with the report that the age-standardized rate and ratio of EP has increased in Italy during recent years, owing to the rising trend of *Chlamydia trachomatis* and *Neisseria gonorrhoeae* infections, and the late sequel of PID, in particular in women who had contracted the infection at an early age [40]. And our findings that Italy held the third highest increasing trend of PID and the highest increasing trend of EP among all the countries and territories, may also enhance this result. Measures have been taken to early diagnose and prevent STIs in Italy. To enhance the surveillance of STIs in Italy, which were common but frequently asymptomatic, and to better understand their epidemiology, a network of 13 clinical microbiology laboratories started the sentinel STI surveillance system in 200916. As for countries with high correlation, PID burden in these countries may have a huge influence on the development of EP burden, and more urgent policies should be implemented in these countries to prevent PID and its sequelae, while for those countries without strong correlation, the EP burden may be due to other reasons apart from PID.

Socio-economic development of regions and countries was one of the most crucial factors for PID and EP burdens addressed in previous studies [36, 41]. We observed a general decreasing trend for the ASPR of PID with SDI at the country level in 2019, while the ASIR of EP decreased before the SDI value of 0.7. Countries with SDI values less than 0.7 were classified as Low, Low-Middle, and Middle SDI. These countries with highest PID and EP burden were mainly from Sub-Saharan Africa region, where the SDI value for all countries was less than 0.7 and the burden on PID and its sequelae is more common [2]. Long-term poverty, decreased levels of medical care and education, lack of sex education, and less prevention of STIs following decreased SDI may contribute to higher rates of both PID and EP. In-expensive screening test should be developed for these countries. While the World Health Organization has promoted the development of vaccines against C. trachomatis and N. gonorrhoeae to prevent PID and its long-term sequelae globally, more available medical resources should take the situation of these countries with lower SDI into consideration [42].

Several limitations should be considered in this study. First, the quality and quantity of the input data used in the DisMod-MR 2.1 model may influence the GBD 2019 estimates. In many areas and countries, there was a lack of or poor-quality of data, and only a small number of countries or territories produced genuine national statistics. Also, burden estimates were largely based on modeled rather than accurate data. Second, the data on the pregnancy rate was unavailable, which made it impossible to calculate the ectopic pregnancy ratio per 1,000 pregnancies. Third, urban–rural discrepancies could not be identified as there was no separate data for urban and rural areas. Furthermore, it is difficult to detect the true frequency because some cases are asymptomatic. However, statistics only reflect cases treated in the hospital or with surgery [43]. As a result, the PID and EP burden might be underestimated.

## Conclusion

In conclusion, PID and EP are public health problems that are highly correlated globally but differ significantly between countries. Although the rate of PID prevalence and EP incidence for reproductive-aged women have decreased, these burdens remain high, especially for those women with *Chlamydia trachomatis* and *Neisseria gonorrhea* infections. Effective interventions and strategies should be established according to the local situation by the policymakers.

### Supplementary Information

**Additional file 1. **

## Data Availability

Data used in this study were derived from the Global Burden of Diseases, Injuries, and Risk Factors Study 2019 (GBD 2019) using the Global Health Data Exchange query tool (http://ghdx. healthdata.org/gbd-results-tool).

## Abbreviations

PID: Pelvic inflammatory disease
STI: Sexually transmitted infection
EP: Ectopic pregnancy
REACH: PID Evaluation and Clinical Health
GBD: Global Burden of Disease
GHDx: Global Health Data Exchange
UI: Uncertain interval
SDI: Socio-demographic index
ASR: Age-standardized rates
EAPC: Estimated annual percentage changes
ASPR: Age-standardized prevalence rate
ASIR: Age-standardized incidence rates
CI: Confidence interval
