# Supplementary material for: Global burden of pelvic inflammatory disease and ectopic pregnancy from 1990 to 2019
Source: BMC Public Health. 2023 Oct 2;23:1894. doi: 10.1186/s12889-023-16663-y (PMC10544469; doi:10.1186/s12889-023-16663-y)
Supplement: Supplementary file 1 — Additional file 1. [file 12889_2023_16663_MOESM1_ESM.docx]

**Supplementary**

Table S1. The Burden and Trends as well as Changing Correlations of Pelvic Inflammatory Disease and Ectopic Pregnancy in 204 Countries and Territories from 1990 to 2019.

| **Characteristics** | **ASPR of PID in 2019 (95% UI)** | **EAPC of ASPR from 1990 to 2019 (95% CI)** | **ASIR of EP in 2019 (95% UI)** | **EAPC of ASIR from 1990 to 2019 (95% CI)** | **Correlation coefficient** | **Socio-demographic index of 2019** |
| --- | --- | --- | --- | --- | --- | --- |
| **Central Europe, Eastern Europe, and Central Asia** | | |  |  |  |  |
| **Central Asia** |  |  |  |  |  |  |
| Armenia | 53.98(32.04-84.34) | -0.6(-0.73--0.48) | 313.82(193.49-472.67) | -1.11(-1.75--0.47) | 0.6 | 0.689 |
| Azerbaijan | 58.27(34.15-92.41) | -0.23(-0.35--0.1) | 301.41(186.03-456.82) | -1.44(-1.84--1.04) | 0.37 | 0.683 |
| Georgia | 87.11(59.9-118.92) | 1.52(1.1-1.94) | 362.28(235.78-522.15) | 1.1(0.69-1.51) | 0.85 | 0.701 |
| Kazakhstan | 57.34(33.52-91.19) | -0.38(-0.54--0.22) | 435.3(266.48-662.18) | 0.64(0.13-1.15) | -0.21 | 0.723 |
| Kyrgyzstan | 62.72(36.95-99.88) | -0.12(-0.25-0.01) | 488.65(303.24-736.11) | 0.01(-0.5-0.52) | -0.1 | 0.596 |
| Mongolia | 59.19(35.21-91.85) | -0.5(-0.65--0.35) | 610.26(372.59-927.55) | -0.24(-1.01-0.54) | 0.48 | 0.606 |
| Tajikistan | 60.36(35.57-94.58) | -0.59(-0.74--0.44) | 584.51(357.14-892.81) | -1.53(-1.83--1.23) | 0.85 | 0.539 |
| Turkmenistan | 57.85(34.-92.19) | -0.51(-0.67--0.36) | 528.75(324.27-791.89) | -1.48(-2.01--0.95) | 0.86 | 0.67 |
| Uzbekistan | 61.13(36.25-96.17) | -0.53(-0.67--0.38) | 381.3(236.22-569.08) | -2.03(-2.54--1.52) | 0.76 | 0.631 |
| Albania | 48.38(28.35-76.33) | -0.74(-0.88--0.61) | 335.43(209.3-494.3) | -1.89(-2.35--1.43) | 0.93 | 0.681 |
| Bosnia and Herzegovina | 46.48(27.56-73.54) | -0.94(-1.1--0.77) | 226.53(139.49-337.66) | -1.28(-1.84--0.71) | 0.88 | 0.718 |
| Bulgaria | 43.79(25.67-69.57) | -0.59(-0.72--0.47) | 272.71(170.54-406.8) | 0.76(0.26-1.25) | -0.29 | 0.764 |
| Croatia | 49.09(30.85-72.37) | 0.64(0.17-1.12) | 248.26(157.55-362.67) | -0.03(-0.4-0.35) | -0.39 | 0.794 |
| Czech Republic | 41.99(24.28-67.33) | -0.57(-0.68--0.47) | 359.05(220.03-538.56) | 0.82(0.15-1.49) | -0.13 | 0.828 |
| Hungary | 43.76(25.91-69.29) | -0.63(-0.76--0.51) | 239.98(147.53-357.62) | -0.35(-0.82-0.11) | 0.5 | 0.791 |
| Montenegro | 44.78(26.08-71.15) | -0.67(-0.77--0.56) | 256.49(156.69-392.15) | -0.82(-1.02--0.62) | 0.74 | 0.791 |
| North Macedonia | 44.25(25.76-69.93) | -0.75(-0.85--0.66) | 240.99(148.47-356.85) | -1.61(-2.07--1.14) | 0.88 | 0.744 |
| Poland | 46.36(31.56-64.76) | -0.15(-0.36-0.05) | 95.24(72.4-122.95) | -0.91(-1.43--0.39) | 0.55 | 0.802 |
| Romania | 45.43(26.54-71.83) | -0.75(-0.88--0.61) | 375.65(233.24-559.58) | 0.39(-0.03-0.81) | 0.02 | 0.76 |
| Serbia | 45.06(26.35-71.35) | 0.22(-0.06-0.5) | 213.84(130.56-322.85) | -1.49(-1.94--1.04) | -0.36 | 0.767 |
| Slovakia | 42.95(24.83-68.46) | 0.2(-0.12-0.53) | 303.6(188.-455.51) | -0.59(-1.33-0.16) | -0.05 | 0.812 |
| Slovenia | 40.95(23.7-65.39) | -0.66(-0.77--0.56) | 252.07(153.37-384.99) | 1.(0.6-1.41) | -0.61 | 0.84 |
| Belarus | 51.65(30.31-81.49) | -0.39(-0.51--0.27) | 652.11(396.74-983.68) | 1.23(0.54-1.91) | -0.26 | 0.745 |
| Estonia | 49.57(28.76-78.43) | -0.53(-0.68--0.37) | 701.85(429.85-1077.11) | 1.3(0.82-1.78) | -0.51 | 0.835 |
| Latvia | 46.87(27.42-74.26) | -0.63(-0.75--0.5) | 771.93(475.17-1175.97) | 1.1(0.46-1.74) | -0.38 | 0.82 |
| Lithuania | 46.66(27.43-73.46) | -0.47(-0.56--0.38) | 571.17(353.69-867.86) | 0.63(-0.07-1.32) | -0.29 | 0.843 |
| Moldova | 54.69(32.52-86.48) | -1.03(-1.2--0.87) | 469.13(284.94-695.23) | -1.27(-1.69--0.85) | 0.55 | 0.696 |
| Russia | 52.55(31.37-81.98) | -0.55(-0.85--0.25) | 680.3(431.7-1015.79) | 1.65(0.96-2.33) | -0.86 | 0.805 |
| Ukraine | 50.65(29.65-80.83) | -0.49(-0.66--0.33) | 596.03(365.12-897.09) | 0.65(-0.07-1.38) | -0.3 | 0.736 |
| **High-income** |  |  |  |  |  |  |
| **Australasia** |  |  |  |  |  |  |
| Australia | 90.25(53.54-143.36) | -0.4(-0.53--0.28) | 60.65(42.26-84.01) | -0.57(-1.2-0.06) | 0.23 | 0.839 |
| New Zealand | 104.34(67.76-152.37) | 0.46(0.33-0.58) | 567.3(374.96-804.24) | -0.1(-0.39-0.2) | -0.31 | 0.84 |
| Brunei | 79.5(47.01-125.29) | -0.51(-0.6--0.42) | 154.98(96.77-232.53) | -2.29(-2.51--2.06) | 0.9 | 0.823 |
| Japan | 85.43(53.31-128.33) | 0.42(0.3-0.54) | 139.63(88.15-208.95) | -0.11(-0.39-0.17) | -0.11 | 0.87 |
| South Korea | 79.74(46.49-125.37) | -0.59(-0.73--0.45) | 87.95(71.66-107.69) | -2.43(-2.88--1.97) | 0.86 | 0.878 |
| Singapore | 76.13(44.54-120.13) | -0.59(-0.68--0.49) | 106.8(65.87-161.74) | -1.54(-2.06--1.01) | 0.54 | 0.861 |
| **High-income North America** | |  |  |  |  |  |
| Canada | 49.58(29.4-78.67) | -0.4(-0.52--0.28) | 223.99(141.2-338.33) | 0.32(0.05-0.59) | -0.36 | 0.873 |
| Greenland | 55.08(32.68-86.97) | -0.48(-0.6--0.36) | 249.45(155.99-372.95) | -0.83(-1.05--0.61) | 0.73 | 0.761 |
| USA | 49.57(34.7-67.93) | -0.71(-0.96--0.46) | 144.23(110.91-187.3) | -2.71(-3.16--2.25) | 0.76 | 0.859 |
| **High-income North America** | |  |  |  |  |  |
| Argentina | 65.92(38.83-104.32) | -0.52(-0.65--0.4) | 708.39(435.57-1065.66) | -0.77(-0.95--0.6) | 0.74 | 0.708 |
| Chile | 63.21(37.32-99.96) | -0.52(-0.63--0.41) | 625.07(386.62-937.62) | -1.18(-1.45--0.91) | 0.83 | 0.759 |
| Uruguay | 70.46(41.91-111.22) | -0.39(-0.54--0.25) | 593.78(367.84-891.69) | -1.01(-1.26--0.76) | 0.67 | 0.697 |
| Western Europe |  |  |  |  |  |  |
| Andorra | 11.29(6.5-18.26) | -0.42(-0.57--0.27) | 291.13(177.53-440.25) | -0.14(-0.29-0.02) | 0.48 | 0.894 |
| Austria | 11.85(6.81-19.22) | -0.35(-0.48--0.22) | 835.8(539.64-1236.73) | -0.06(-0.37-0.25) | 0.43 | 0.849 |
| Belgium | 9.96(5.29-17.04) | 1.88(0.35-3.43) | 535.51(334.26-792.61) | 0.39(0.17-0.6) | 0.12 | 0.851 |
| Cyprus | 6.85(3.38-12.08) | 2.04(0.94-3.15) | 187.86(115.99-284.65) | -2.92(-3.27--2.57) | -0.55 | 0.841 |
| Denmark | 11.33(6.52-18.36) | -0.55(-0.68--0.41) | 485.39(296.19-717.99) | 0.24(0.02-0.45) | -0.32 | 0.89 |
| Finland | 14.79(8.4-24.04) | -0.31(-1.32-0.72) | 372.9(228.55-565.06) | -0.68(-0.94--0.42) | 0.45 | 0.856 |
| France | 11.29(6.44-18.39) | -0.4(-0.51--0.29) | 431.91(266.91-652.61) | 0.44(0.19-0.68) | -0.48 | 0.834 |
| Germany | 10.7(6.14-17.14) | -0.57(-0.7--0.44) | 160.77(131.81-192.52) | 0.91(0.61-1.2) | -0.74 | 0.898 |
| Greece | 11.39(6.55-18.28) | -0.53(-0.64--0.42) | 315.97(192.76-485.43) | 0.79(0.46-1.13) | -0.49 | 0.794 |
| Iceland | 5.17(2.63-9.07) | 0.46(-0.42-1.34) | 360.6(219.35-549.06) | -0.65(-0.91--0.38) | -0.07 | 0.869 |
| Ireland | 11.59(6.66-18.49) | -0.6(-0.73--0.48) | 447.2(274.01-673.36) | -0.02(-0.23-0.19) | 0.16 | 0.867 |
| Israel | 11.29(6.53-18.24) | -0.58(-0.71--0.44) | 737.65(452.05-1123.43) | 0.5(0.25-0.75) | -0.43 | 0.803 |
| Italy | 61.55(38.88-91.18) | 1.85(1.22-2.48) | 273.6(175.21-394.37) | 1.67(1.29-2.06) | 0.89 | 0.801 |
| Luxembourg | 15.12(8.79-24.17) | -0.19(-1.33-0.95) | 431.32(264.2-644.17) | -0.18(-0.4-0.03) | -0.04 | 0.895 |
| Malta | 7.92(3.86-14.14) | 1.51(-0.14-3.19) | 164.84(101.2-250.05) | -1.04(-1.69--0.38) | -0.89 | 0.801 |
| Monaco | 11.32(6.45-18.14) | -0.42(-0.53--0.3) | 358.24(219.68-539.01) | -0.21(-0.47-0.05) | 0.22 | 0.902 |
| Netherlands | 11.74(6.77-18.95) | -0.64(-0.79--0.49) | 400.01(246.07-604.19) | 0.35(0.1-0.61) | -0.33 | 0.883 |
| Norway | 44.19(26.18-69.74) | 1.1(0.65-1.55) | 305.79(156.23-532.62) | -0.12(-0.35-0.12) | 0.21 | 0.913 |
| Portugal | 6.94(4.12-10.83) | 0.35(-0.33-1.04) | 191.46(123.36-274.44) | -0.33(-0.53--0.12) | 0 | 0.743 |
| San Marino | 10.69(6.19-17.36) | -0.44(-0.57--0.32) | 357.9(220.68-533.92) | 0.12(-0.09-0.34) | -0.14 | 0.884 |
| Spain | 12.77(7.38-20.51) | -0.53(-0.67--0.39) | 306.1(189.17-465.44) | 0.42(0.19-0.65) | -0.38 | 0.767 |
| Sweden | 27.55(16.28-43.51) | 1.22(0.43-2.01) | 617.88(384.75-936.24) | -0.5(-0.88--0.12) | -0.87 | 0.872 |
| Switzerland | 16.07(9.32-25.69) | -0.21(-1.27-0.85) | 406.99(248.96-614.24) | 0.33(0.-0.67) | -0.56 | 0.929 |
| UK | 66.41(41.01-100.76) | 0.78(0.29-1.26) | 443.78(281.96-650.15) | 0.36(0.09-0.63) | 0.05 | 0.847 |
| **Latin America and Caribbean** | |  |  |  |  |  |
| **Andean Latin America** | |  |  |  |  |  |
| Bolivia | 70.88(42.11-110.49) | -0.56(-0.7--0.41) | 706.2(434.21-1065.89) | -1.41(-1.63--1.2) | 0.9 | 0.566 |
| Ecuador | 71.2(53.-93.21) | 0.03(-0.17-0.22) | 487.99(320.94-693.96) | -0.44(-0.74--0.14) | -0.19 | 0.64 |
| Peru | 69.1(40.9-109.66) | -0.53(-0.65--0.41) | 477.75(296.1-729.2) | -1.74(-1.94--1.55) | 0.92 | 0.648 |
| Caribbean |  |  |  |  |  |  |
| Antigua and Barbuda | 46.34(27.08-73.13) | -0.44(-0.55--0.32) | 174.75(107.03-263.85) | -1.44(-1.62--1.25) | 0.84 | 0.743 |
| The Bahamas | 48.35(28.01-76.85) | -0.49(-0.63--0.35) | 173.93(106.28-261.95) | -1.74(-2.--1.49) | 0.72 | 0.796 |
| Barbados | 50.41(29.63-79.2) | -0.35(-0.46--0.24) | 163.14(100.39-247.98) | -0.75(-0.96--0.55) | 0.59 | 0.742 |
| Belize | 54.18(31.88-85.53) | -0.54(-0.64--0.44) | 252.17(154.94-380.27) | -2.59(-2.75--2.43) | 0.9 | 0.603 |
| Bermuda | 43.82(25.49-69.35) | -0.58(-0.7--0.46) | 180.5(110.86-276.8) | -0.06(-0.32-0.19) | 0.06 | 0.813 |
| Cuba | 59.63(35.51-92.53) | 0.34(-0.15-0.83) | 169.73(103.31-257.73) | 0.46(0.16-0.75) | 0.14 | 0.668 |
| Dominica | 47.44(27.8-76.18) | -0.63(-0.74--0.51) | 212.9(130.62-320.24) | -2.05(-2.24--1.85) | 0.91 | 0.729 |
| Dominican Republic | 51.05(30.24-80.42) | -0.63(-0.78--0.49) | 279.(170.55-415.32) | -1.74(-2.06--1.41) | 0.89 | 0.592 |
| Grenada | 52.89(30.89-84.16) | -0.56(-0.69--0.42) | 243.25(149.59-366.13) | -1.59(-1.94--1.24) | 0.86 | 0.669 |
| Guyana | 55.64(32.8-87.12) | -0.49(-0.61--0.37) | 244.6(150.34-368.76) | -1.23(-1.37--1.08) | 0.83 | 0.618 |
| Haiti | 57.81(35.43-88.65) | -0.94(-1.08--0.8) | 435.51(260.68-671.47) | -1.97(-2.11--1.84) | 0.92 | 0.432 |
| Jamaica | 51.75(30.54-81.29) | -0.47(-0.61--0.34) | 191.14(115.59-291.38) | -1.8(-1.96--1.64) | 0.8 | 0.684 |
| Puerto Rico | 47.74(28.05-75.01) | -0.55(-0.68--0.43) | 111.65(68.97-168.47) | -2.58(-2.87--2.28) | 0.85 | 0.814 |
| Saint Kitts and Nevis | 45.49(26.78-72.19) | -0.53(-0.68--0.38) | 182.61(112.23-276.16) | -1.82(-2.--1.63) | 0.81 | 0.746 |
| Saint Lucia | 52.88(31.-85.05) | -0.61(-0.73--0.49) | 169.44(104.85-255.44) | -2.46(-2.71--2.21) | 0.87 | 0.67 |
| Saint Vincent and the Grenadines | 54.56(31.78-87.68) | -0.56(-0.69--0.43) | 211.38(128.9-320.48) | -1.42(-1.68--1.16) | 0.84 | 0.627 |
| Suriname | 52.97(31.32-83.18) | -0.48(-0.59--0.37) | 263.14(160.92-395.2) | -0.88(-1.14--0.63) | 0.76 | 0.636 |
| Trinidad and Tobago | 49.04(28.95-77.83) | -0.6(-0.72--0.47) | 192.28(118.86-285.53) | -0.78(-1.14--0.43) | 0.56 | 0.757 |
| Virgin Islands | 44.04(25.61-70.84) | -0.64(-0.75--0.52) | 206.45(126.19-311.46) | -1.78(-1.99--1.57) | 0.85 | 0.799 |
| **Central Latin America** | |  |  |  |  |  |
| Colombia | 35.63(20.82-56.36) | -0.86(-0.99--0.73) | 273.84(169.52-413.45) | -1.96(-2.11--1.8) | 0.94 | 0.633 |
| Costa Rica | 31.5(18.17-50.47) | -0.77(-0.89--0.65) | 224.19(138.84-338.07) | -2.1(-2.49--1.7) | 0.86 | 0.68 |
| El Salvador | 33.99(19.87-53.55) | -0.97(-1.11--0.83) | 254.7(155.22-386.43) | -2.36(-2.75--1.97) | 0.92 | 0.573 |
| Guatemala | 37.81(22.14-59.94) | -0.97(-1.09--0.85) | 362.94(222.46-554.02) | -3.57(-3.87--3.28) | 0.94 | 0.526 |
| Honduras | 38.17(22.73-58.54) | -0.84(-1.02--0.66) | 334.8(206.33-510.74) | -3.17(-3.42--2.92) | 0.77 | 0.496 |
| Mexico | 44.7(27.91-66.5) | -0.03(-0.24-0.17) | 317.3(206.53-459.45) | -1.9(-2.3--1.49) | 0.35 | 0.649 |
| Nicaragua | 37.7(21.84-59.53) | -0.86(-1.02--0.69) | 297.39(185.61-442.4) | -2.65(-3.13--2.17) | 0.95 | 0.517 |
| Panama | 32.98(19.23-52.09) | -0.32(-0.46--0.18) | 315.31(194.15-476.26) | -0.46(-0.69--0.23) | 0.5 | 0.686 |
| Venezuela | 33.93(19.75-54.28) | -0.83(-0.96--0.71) | 261.9(160.42-400.94) | -1.63(-1.91--1.36) | 0.88 | 0.607 |
| **Tropical Latin America** | |  |  |  |  |  |
| Brazil | 50.53(32.01-75.06) | -1.45(-1.78--1.12) | 171.76(111.38-251.78) | -1.11(-1.38--0.85) | 0.57 | 0.64 |
| Paraguay | 58.53(34.95-91.75) | -0.74(-0.85--0.63) | 163.87(102.72-246.36) | -2.35(-2.55--2.15) | 0.93 | 0.638 |
| **North Africa and Middle East** | |  |  |  |  |  |
| **North Africa and Middle East** | |  |  |  |  |  |
| Afghanistan | 78.51(46.69-123.6) | 0.1(-0.1-0.31) | 556.42(345.68-836.5) | -1.14(-1.28--0.99) | -0.33 | 0.343 |
| Algeria | 63.39(37.66-99.62) | -0.35(-0.48--0.21) | 341.2(211.84-511.99) | -0.52(-1.13-0.1) | 0.61 | 0.652 |
| Bahrain | 55.95(33.24-87.72) | -0.32(-0.46--0.18) | 164.86(101.81-247.53) | -2.59(-2.87--2.31) | 0.67 | 0.751 |
| Egypt | 64.68(38.44-101.24) | -0.21(-0.37--0.05) | 265.88(166.12-400.91) | -2.24(-2.92--1.57) | 0.68 | 0.658 |
| Iran | 58.06(34.67-91.46) | 0.16(-0.11-0.43) | 99.1(61.78-150.57) | -2.52(-3.04--2.) | -0.5 | 0.67 |
| Iraq | 64.35(37.66-102.21) | -0.46(-0.63--0.29) | 278.69(172.12-418.77) | -3.16(-3.29--3.03) | 0.77 | 0.671 |
| Jordan | 59.7(35.66-94.24) | -0.45(-0.57--0.33) | 434.71(278.06-624.13) | -1.69(-1.96--1.43) | 0.82 | 0.731 |
| Kuwait | 56.64(33.22-90.35) | -0.41(-0.59--0.23) | 96.42(59.89-144.91) | -3.89(-4.75--3.03) | 0.58 | 0.851 |
| Lebanon | 61.41(35.87-97.37) | -0.42(-0.55--0.3) | 209.69(130.63-313.71) | -2.61(-2.8--2.41) | 0.8 | 0.708 |
| Libya | 56.42(33.3-89.72) | -0.46(-0.65--0.27) | 184.56(115.65-270.81) | -3.2(-3.41--2.99) | 0.77 | 0.709 |
| Morocco | 70.56(41.36-112.09) | -0.16(-0.32-0.) | 244.58(152.02-365.18) | -2.28(-2.41--2.15) | 0.36 | 0.548 |
| Oman | 54.44(32.2-85.89) | -0.89(-1.06--0.72) | 234.03(145.67-351.79) | -4.3(-4.55--4.06) | 0.92 | 0.783 |
| Palestine | 62.64(37.-99.25) | -0.77(-0.91--0.63) | 242.94(150.8-364.04) | -3.55(-3.8--3.31) | 0.89 | 0.588 |
| Qatar | 56.21(33.12-88.89) | -0.53(-0.68--0.38) | 218.2(135.24-323.58) | -2.72(-2.88--2.56) | 0.84 | 0.83 |
| Saudi Arabia | 53.29(31.43-84.23) | -0.88(-1.06--0.7) | 238.85(148.98-363.07) | -3.58(-3.72--3.45) | 0.89 | 0.805 |
| Sudan | 70.97(42.16-110.48) | -0.32(-0.5--0.13) | 355.66(221.7-528.44) | -2.51(-2.68--2.34) | 0.52 | 0.515 |
| Syria | 66.52(38.92-104.9) | -0.45(-0.62--0.27) | 206.78(128.5-311.54) | -2.9(-3.4--2.4) | 0.54 | 0.619 |
| Tunisia | 58.4(34.42-92.21) | -0.45(-0.58--0.32) | 206.69(128.87-307.13) | -1.84(-2.15--1.53) | 0.88 | 0.672 |
| Turkey | 57.56(33.63-91.83) | -0.29(-0.5--0.08) | 275.26(167.89-413.42) | -1.7(-1.94--1.45) | 0.39 | 0.748 |
| United Arab Emirates | 53.57(31.39-85.63) | -0.37(-0.52--0.22) | 133.5(83.22-199.08) | -3.69(-3.85--3.53) | 0.79 | 0.88 |
| Yemen | 73.41(43.54-115.41) | -0.43(-0.59--0.26) | 406.78(251.04-612.21) | -2.63(-2.82--2.44) | 0.67 | 0.412 |
| **South Asia** | |  |  |  |  |  |
| **South Asia** |  |  |  |  |  |  |
| Bangladesh | 73.78(43.6-114.6) | -2.55(-3.04--2.05) | 209.52(128.24-313.87) | -3.14(-3.36--2.91) | 0.94 | 0.483 |
| Bhutan | 72.78(42.97-113.72) | -0.86(-0.98--0.75) | 248.79(148.15-377.14) | -3.41(-3.55--3.28) | 0.95 | 0.455 |
| India | 66.93(40.86-102.07) | -0.75(-0.89--0.6) | 213.73(132.04-315.87) | -3.06(-3.21--2.91) | 0.91 | 0.566 |
| Nepal | 88.28(58.75-126.5) | 0.09(-0.25-0.43) | 239.76(146.21-360.1) | -4.35(-4.64--4.06) | 0.06 | 0.422 |
| Pakistan | 69.87(41.02-110.11) | -0.47(-0.67--0.27) | 509.27(319.06-755.77) | -2.21(-2.39--2.03) | 0.62 | 0.449 |
| **Southeast Asia, East Asia, and Oceania** | | |  |  |  |  |
| **East Asia** |  |  |  |  |  |  |
| China | 37.89(23.56-56.7) | -0.28(-0.59-0.02) | 386.52(253.66-552.68) | -0.76(-1.4--0.11) | 0.54 | 0.686 |
| North Korea | 35.63(21.22-55.8) | -0.65(-0.81--0.5) | 419.62(259.69-622.6) | -3.31(-3.59--3.03) | 0.88 | 0.558 |
| Taiwan (Province of China) | 27.8(16.36-43.83) | -0.9(-1.05--0.75) | 347.25(219.34-511.59) | -0.77(-1.37--0.16) | 0.43 | 0.868 |
| **Oceania** |  |  |  |  |  |  |
| American Samoa | 47.29(28.07-73.47) | -0.41(-0.62--0.21) | 472.44(291.2-703.82) | -1.77(-1.92--1.62) | 0.62 | 0.712 |
| Cook Islands | 50.67(29.4-79.94) | -0.43(-0.63--0.22) | 399.25(246.52-600.49) | -1.45(-1.59--1.3) | 0.62 | 0.764 |
| Fiji | 59.13(34.78-91.9) | -0.55(-0.8--0.3) | 434.25(264.61-663.88) | -0.32(-0.48--0.17) | 0.37 | 0.664 |
| Guam | 43.93(26.2-68.48) | -0.4(-0.57--0.23) | 507.07(313.61-761.22) | -0.1(-0.43-0.22) | 0.3 | 0.813 |
| Kiribati | 66.04(39.05-102.1) | -0.07(-0.28-0.13) | 580.56(358.57-879.42) | -0.97(-1.14--0.81) | 0.11 | 0.527 |
| Marshall Islands | 54.61(32.16-85.44) | -0.38(-0.56--0.2) | 422.93(257.-638.89) | -1.46(-1.65--1.27) | 0.6 | 0.544 |
| Federated States of Micronesia | 59.17(35.04-91.92) | -0.43(-0.61--0.25) | 450.02(271.95-679.41) | -2.62(-2.77--2.47) | 0.72 | 0.58 |
| Nauru | 56.37(33.07-88.08) | -0.19(-0.42-0.05) | 593.33(362.38-892.29) | -1.99(-2.14--1.84) | 0.19 | 0.618 |
| Niue | 52.61(30.9-82.42) | -0.34(-0.52--0.15) | 426.45(256.35-642.5) | -1.67(-1.82--1.53) | 0.63 | 0.711 |
| Northern Mariana Islands | 46.95(27.6-74.24) | -0.2(-0.4--0.01) | 355.25(219.47-531.52) | -0.44(-0.65--0.23) | 0.3 | 0.771 |
| Palau | 52.5(30.83-82.05) | -0.32(-0.5--0.13) | 352.(213.9-529.57) | -1.29(-1.47--1.11) | 0.64 | 0.738 |
| Papua New Guinea | 94.92(63.04-137.83) | -1.37(-1.64--1.09) | 820.41(504.11-1229.99) | -0.6(-0.72--0.49) | 0.81 | 0.394 |
| Samoa | 59.77(35.33-93.97) | -0.13(-0.33-0.06) | 230.03(140.37-348.85) | -2.32(-2.5--2.14) | 0.24 | 0.641 |
| Solomon Islands | 70.39(42.04-108.17) | -0.58(-0.82--0.34) | 792.71(482.53-1198.33) | -1.62(-1.77--1.48) | 0.67 | 0.407 |
| Tokelau | 51.(29.87-80.58) | -0.58(-0.76--0.4) | 762.71(474.75-1162.39) | -1.35(-1.48--1.21) | 0.78 | 0.626 |
| Tonga | 55.62(32.53-86.94) | -0.34(-0.52--0.16) | 672.7(417.54-1006.21) | -0.53(-0.65--0.4) | 0.61 | 0.636 |
| Tuvalu | 52.4(30.97-82.09) | -0.42(-0.61--0.23) | 485.64(298.77-728.34) | -1.7(-1.85--1.54) | 0.68 | 0.589 |
| Vanuatu | 56.47(33.38-87.6) | -0.47(-0.66--0.27) | 548.88(336.79-825.04) | -1.81(-1.93--1.68) | 0.65 | 0.485 |
| **Southeast Asia** |  |  |  |  |  |  |
| Cambodia | 20.82(12.19-32.85) | -1.15(-1.32--0.98) | 246.61(151.53-372.4) | -3.47(-3.75--3.2) | 0.94 | 0.469 |
| Indonesia | 11.32(6.93-17.35) | -1.6(-1.77--1.43) | 162.94(104.6-239.84) | -1.43(-1.61--1.24) | 0.92 | 0.66 |
| Laos | 20.92(12.34-33.2) | -1.29(-1.44--1.13) | 242.1(148.8-365.27) | -3.16(-3.4--2.92) | 0.95 | 0.49 |
| Malaysia | 17.41(10.14-27.34) | -1.32(-1.56--1.09) | 233.05(146.34-353.36) | -2.01(-2.28--1.73) | 0.8 | 0.737 |
| Maldives | 19.3(11.31-30.74) | -0.86(-0.99--0.72) | 210.95(128.96-317.19) | -2.8(-3.21--2.38) | 0.89 | 0.562 |
| Mauritius | 16.43(9.65-26.04) | -0.55(-0.69--0.42) | 139.74(86.85-212.11) | -1.58(-1.84--1.32) | 0.75 | 0.705 |
| Myanmar | 20.94(12.39-32.99) | -2.19(-2.42--1.97) | 224.46(137.96-340.09) | -2.04(-2.17--1.91) | 0.93 | 0.521 |
| Philippines | 30.79(18.17-48.68) | 1.35(1.03-1.67) | 391.42(244.79-580.63) | -1.56(-1.7--1.41) | -0.89 | 0.623 |
| Seychelles | 17.77(10.32-28.38) | -0.35(-0.5--0.21) | 209.18(128.67-317.44) | -0.32(-0.69-0.05) | 0.2 | 0.724 |
| Sri Lanka | 16.76(9.87-26.63) | -0.67(-0.83--0.51) | 194.75(119.41-295.4) | -0.55(-0.8--0.3) | 0.71 | 0.69 |
| Thailand | 17.63(10.29-27.93) | -0.84(-1.16--0.53) | 98.92(61.24-148.54) | -1.74(-2.01--1.48) | 0.51 | 0.687 |
| Timor-Leste | 20.66(12.1-32.82) | -0.87(-0.99--0.76) | 452.77(280.41-683.28) | -1.75(-2.04--1.45) | 0.9 | 0.514 |
| Vietnam | 25.5(16.22-36.96) | 0.6(0.25-0.95) | 152.48(94.53-229.17) | -1.78(-1.98--1.58) | -0.42 | 0.617 |
| **Sub-Saharan Africa** | |  |  |  |  |  |
| **Central Sub-Saharan Africa** | |  |  |  |  |  |
| Angola | 82.58(50.86-125.33) | -1.42(-1.62--1.22) | 574.75(343.82-878.35) | -1.28(-1.45--1.11) | 0.85 | 0.47 |
| Central African Republic | 93.33(58.14-139.79) | -0.17(-0.35-0.02) | 550.13(331.28-831.83) | -0.95(-1.06--0.84) | 0.19 | 0.274 |
| Congo (Brazzaville) | 77.15(48.07-117.72) | -0.74(-0.88--0.6) | 398.92(238.32-607.12) | -1.52(-1.7--1.33) | 0.85 | 0.568 |
| DR Congo | 83.81(51.07-127.67) | -0.34(-0.47--0.21) | 557.75(335.42-854.1) | -1.35(-1.6--1.09) | 0.61 | 0.382 |
| Equatorial Guinea | 71.78(42.41-113.61) | -1.41(-1.56--1.26) | 399.03(241.91-600.) | -3.12(-3.35--2.89) | 0.94 | 0.685 |
| Gabon | 73.28(43.97-115.63) | -0.41(-0.56--0.27) | 326.91(196.28-496.64) | -2.58(-2.68--2.48) | 0.78 | 0.656 |
| **Eastern Sub-Saharan Africa** | |  |  |  |  |  |
| Burundi | 85.76(52.78-132.7) | -0.5(-0.75--0.25) | 751.69(451.06-1140.21) | -0.56(-0.67--0.45) | 0.38 | 0.284 |
| Comoros | 75.43(44.66-117.53) | -0.28(-0.41--0.14) | 363.08(218.54-547.22) | -2.79(-2.9--2.68) | 0.63 | 0.455 |
| Djibouti | 76.1(45.35-118.2) | -0.37(-0.47--0.27) | 459.45(275.48-696.31) | -2.23(-2.43--2.03) | 0.77 | 0.459 |
| Eritrea | 74.71(45.78-114.05) | -0.77(-1.01--0.53) | 487.16(292.01-744.04) | -1.76(-1.92--1.6) | 0.68 | 0.396 |
| Ethiopia | 73.(44.31-113.05) | -2.22(-2.59--1.85) | 586.71(356.57-884.29) | -1.68(-1.79--1.57) | 0.9 | 0.343 |
| Kenya | 65.95(39.59-102.72) | -0.38(-0.51--0.26) | 368.31(227.25-551.57) | -2.62(-2.75--2.49) | 0.82 | 0.508 |
| Madagascar | 75.48(46.35-114.85) | -0.81(-0.99--0.63) | 437.91(263.69-664.67) | -1.82(-1.95--1.69) | 0.84 | 0.396 |
| Malawi | 80.72(49.24-124.04) | -1.04(-1.26--0.82) | 462.82(275.96-711.4) | -2.15(-2.37--1.93) | 0.84 | 0.384 |
| Mozambique | 79.61(48.99-121.31) | -1.39(-1.64--1.14) | 572.52(345.45-874.45) | -0.9(-1.04--0.75) | 0.72 | 0.307 |
| Rwanda | 73.7(44.33-114.77) | -1.5(-1.76--1.24) | 427.4(259.08-647.42) | -2.65(-2.85--2.45) | 0.92 | 0.429 |
| Somalia | 95.09(59.69-141.12) | -0.41(-0.64--0.18) | 835.61(504.06-1262.06) | -0.48(-0.54--0.43) | 0.48 | 0.081 |
| South Sudan | 82.81(51.84-123.85) | -1.03(-1.22--0.84) | 668.96(397.75-1016.91) | -0.38(-0.45--0.31) | 0.74 | 0.363 |
| Uganda | 74.31(44.8-115.49) | -0.68(-0.88--0.49) | 576.85(346.09-864.98) | -1.67(-1.86--1.48) | 0.72 | 0.404 |
| Tanzania | 76.3(46.21-118.55) | -0.6(-0.79--0.42) | 535.53(325.23-813.74) | -1.12(-1.24--1.) | 0.68 | 0.423 |
| Zambia | 70.18(42.96-107.63) | -1.9(-2.11--1.69) | 481.38(290.73-732.21) | -1.82(-1.97--1.68) | 0.92 | 0.505 |
| **Southern Sub-Saharan Africa** | |  |  |  |  |  |
| Botswana | 85.51(51.55-133.28) | -0.22(-0.4--0.04) | 597.73(361.91-895.97) | -1.89(-1.98--1.79) | 0.51 | 0.634 |
| Eswatini | 90.18(53.77-141.49) | -0.2(-0.33--0.07) | 286.11(172.3-430.28) | -2.22(-2.32--2.12) | 0.57 | 0.577 |
| Lesotho | 87.59(52.47-136.19) | -0.05(-0.2-0.1) | 266.38(160.31-403.45) | -2.11(-2.26--1.97) | 0.29 | 0.507 |
| Namibia | 80.3(48.32-125.37) | -0.56(-0.68--0.44) | 296.88(177.45-448.2) | -1.56(-1.68--1.44) | 0.87 | 0.612 |
| South Africa | 74.19(44.81-115.24) | -1.98(-2.57--1.37) | 75.01(46.84-111.16) | -2.3(-2.51--2.09) | 0.68 | 0.678 |
| Zimbabwe | 94.08(57.35-145.32) | 0.34(0.19-0.48) | 352.38(210.77-536.79) | -1.06(-1.24--0.88) | -0.44 | 0.476 |
| **Western Sub-Saharan Africa** | |  |  |  |  |  |
| Benin | 130.17(79.66-199.24) | -0.87(-1.21--0.52) | 619.74(373.88-946.13) | -0.89(-1.--0.79) | 0.67 | 0.352 |
| Burkina Faso | 133.2(83.07-199.69) | -2.54(-2.96--2.1) | 674.31(405.03-1024.39) | -1.02(-1.09--0.95) | 0.96 | 0.257 |
| Cape Verde | 126.84(76.2-199.92) | 0.03(-0.15-0.21) | 244.64(148.18-375.28) | -2.88(-3.04--2.72) | -0.07 | 0.525 |
| Cameroon | 122.24(75.71-185.73) | -2.63(-2.95--2.3) | 437.35(263.09-666.74) | -1.47(-1.75--1.2) | 0.76 | 0.49 |
| Chad | 127.04(80.79-188.26) | -1.53(-1.84--1.22) | 813.01(489.45-1234.29) | -0.38(-0.46--0.31) | 0.84 | 0.238 |
| Ivory Coast | 122.23(75.69-185.04) | -1.75(-2.07--1.43) | 524.53(316.26-800.64) | -1.5(-1.55--1.44) | 0.91 | 0.408 |
| The Gambia | 133.06(81.56-204.25) | -0.43(-0.69--0.17) | 510.84(310.56-774.11) | -1.87(-2.04--1.7) | 0.45 | 0.399 |
| Ghana | 111.36(68.4-171.48) | -0.51(-0.66--0.35) | 381.49(230.78-579.66) | -1.84(-1.99--1.69) | 0.78 | 0.557 |
| Guinea | 129.15(80.95-191.9) | -1.78(-2.18--1.39) | 541.94(327.4-825.53) | -1.5(-1.56--1.44) | 0.89 | 0.325 |
| Guinea-Bissau | 129.22(78.37-197.63) | -1.7(-1.94--1.46) | 516.47(309.36-782.32) | -1.56(-1.68--1.43) | 0.89 | 0.355 |
| Liberia | 120.72(74.24-184.09) | -1.79(-2.09--1.5) | 410.2(246.47-618.88) | -2.36(-2.53--2.18) | 0.89 | 0.37 |
| Mali | 128.44(80.37-191.98) | -3.51(-3.93--3.09) | 724.74(440.68-1106.4) | -0.87(-0.94--0.8) | 0.92 | 0.263 |
| Mauritania | 122.96(74.9-187.93) | -1.77(-2.11--1.42) | 468.17(284.63-712.31) | -1.72(-1.9--1.53) | 0.81 | 0.496 |
| Niger | 133.56(82.82-204.13) | -1.53(-1.95--1.12) | 871.33(521.99-1352.27) | -0.32(-0.4--0.24) | 0.55 | 0.162 |
| Nigeria | 110.69(67.17-172.49) | -1.81(-2.08--1.54) | 637.38(393.79-954.74) | -1.28(-1.41--1.14) | 0.86 | 0.515 |
| Sao Tome and Principe | 122.6(73.89-193.88) | -0.33(-0.5--0.17) | 350.25(213.6-531.18) | -2.66(-2.84--2.49) | 0.59 |  |
| Senegal | 120.37(74.48-180.8) | -2.39(-2.76--2.02) | 503.69(302.08-763.72) | -1.48(-1.61--1.34) | 0.89 | 0.389 |
| Sierra Leone | 131.61(81.7-198.56) | -0.89(-1.24--0.54) | 486.83(295.6-741.83) | -1.45(-1.61--1.29) | 0.63 | 0.347 |
| Togo | 115.39(71.61-175.18) | -1.61(-1.94--1.28) | 472.81(283.96-726.52) | -1.58(-1.76--1.41) | 0.78 | 0.417 |

*ASPR: age-standardized prevalence rate; PID: pelvic inflammatory disease; ASIR: age-standardized incidence rate; EP: ectopic pregnancy; UI, uncertain interval; CI, confidential interval.


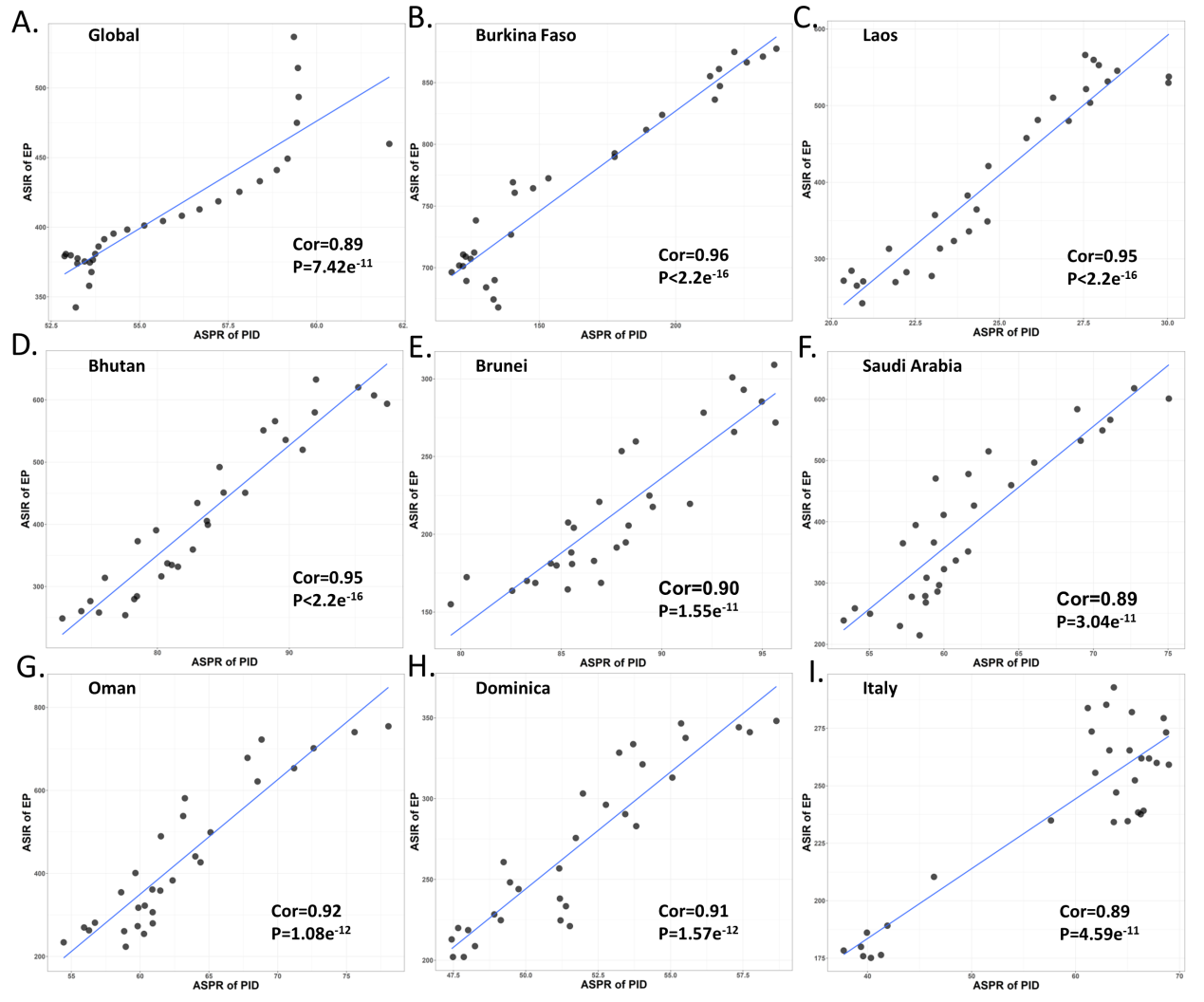


Figure S1. The Correlation between Age-standardized Prevalence Rates (ASPR) of Pelvic Inflammatory Disease (PID) and Age-standardized Incidence Rates of Ectopic Pregnancy (EP) for the Global Level and Some Typical Countries from 1990 to 2019. A) global, B) Burkina Faso, C) Laos, D) Bhutan, E) Brunei, F) Saudi Arabia, G)Oman, H) Dominica, and I) Italy.
